# Real-life effectiveness of anti-malarial treatment regimens: what are we aiming for?

**DOI:** 10.1186/s12936-023-04606-2

**Published:** 2023-06-20

**Authors:** Dorothea Ekoka Mbassi, Christoph Pfaffendorf, Ghyslain Mombo-Ngoma, Benno Kreuels, Michael Ramharter

**Affiliations:** 1 Department of Tropical Medicine, Bernhard Nocht Institute for Tropical Medicine, Hamburg, Germany; 2 Department of Medicine, University Medical Centre Hamburg-Eppendorf, Hamburg, Germany; 3 German Centre for Infection Research (DZIF), Partner Site Hamburg-Lübeck-Borstel, Hamburg, Germany; 4 Department of Implementation Research, Bernhard Nocht Institute for Tropical Medicine, Hamburg, Germany; 5 Centre de Recherches Médicales de Lambaréné, Lambaréné, Gabon; 6 Department of Clinical Pharmacy, Institute of Pharmacy, University of Hamburg, 20146 Hamburg, Germany

**Keywords:** Malaria, Antimalarials, Single-dose, Treatment schedule, Tolerability, Drug development, Single-day

## Abstract

Three-day artemisinin-based combination therapy (ACT) is the current standard of care for the treatment of malaria. However, specific drug resistance associated with reduced efficacy of ACT has been observed, therefore necessitating the clinical development of new anti-malarial drugs and drug combinations. Previously, Single Encounter Radical Cure and Prophylaxis (SERCAP) has been proposed as ideal target-product-profile for any new anti-malarial drug regimen as this would improve treatment adherence besides ensuring complete cure and prevention of early reinfection. Arguably, this concept may not be ideal as it (1) necessitates administration of an excessively high dose of drug to achieve plasmodicidal plasma levels for a sufficient time span, (2) increases the risk for drug related adverse drug reactions, and (3) leaves the patient with a one-time opportunity to achieve—or not—cure by a single drug intake. Over the past years, SERCAP has led to the halt of promising drug development programmes, leading to potentially unnecessary attrition in the anti-malarial development pipeline. One proposition could be the concept of single-day multi-dose regimens as a potentially better alternative, as this allows to (1) administer a lower dose of the drug at each time-point leading to better tolerability and safety, (2) increase treatment adherence based on the intake of the anti-malarial drug within 24 h when malaria-related symptoms are still present, and (3) have more than one opportunity for adequate intake of the drug in case of early vomiting or other factors causing reduced bioavailability. In line with a recently published critical viewpoint on the concept of SERCAP, an alternative proposition is—in contrast to the current World Health Organization (WHO) treatment guidelines—to aim for less than three days, but still multiple-dose anti-malarial treatment regimens. This may help to strike the optimal balance between improving treatment adherence, maximizing treatment effectiveness, while keeping attrition of new drugs and drug regimens as low as possible.

## Background

Artemisinin-based combination therapy (ACT), given for three days, has been the recommended treatment for malaria for the past 20 years. However, ACT is less efficacious in real-world practice than in research settings. This is due to multiple factors such as incomplete administration or non-adherence to the prescribed treatment regimen. Even when knowledge and resources are available, the rapid therapeutic response or the occurrence of treatment-associated adverse drug reactions may lead to premature termination of intake of prescribed treatment regimens [1–4]. While ACT is still highly efficacious in sub-Saharan Africa, clinically relevant emergence of molecular resistance markers and delays in parasitological response have been reported [5]. There is thus a vital need for new effective treatments that are safe, well-tolerated and are characterized by simple and short regimens.

Based on this understanding, the concept of single-dose therapy for uncomplicated malaria became the ultimate goal of clinical drug development of new anti-malarial drug candidates [6]. Single-exposure radical cure and prophylaxis (SERCAP) has been the agreed target product profile in anti-malarial drug development over the past decade [7, 8]. Exemplarily, the PAMAfrica consortium, one of the largest joint efforts of anti-malarial drug development, envisages a single-dose multi-drug combination out of the largest anti-malarial drug portfolio worldwide, coordinated by Medicines for Malaria Venture (MMV) [9]. Similarly, the SINDOFO consortium specifically aims to develop a single-dose therapy from a non-ACT drug class [10]. SERCAP is meant to overcome the problem of sub-optimal treatment adherence by the administration of only a single dose of the anti-malarial.

In his recent opinion article, Professor White argues that the expectations for new drug candidates to fulfil SERCAP criteria are too high and are, therefore, potentially counterproductive for the development of new anti-malarial therapies – a criticism that can be concurred with. A single-dose cure is in itself a pharmacological challenge as the administered dose needs to be excessively high to achieve curative plasma drug concentrations over a sufficient time, accounting for inter-individual differences in bioavailability, ensuring clearance of parasites in patients without significant semi-immunity, such as children, without posing any relevant safety concerns. Contracting a usual three-day treatment course in a single drug administration necessarily increases the risk for treatment associated adverse drug reactions, such as early vomiting, thus leaving the patient potentially without adequate blood concentrations to clear infection [11]. Indeed, while overall ensuring increasing quality of new drug candidates, the concept of SERCAP might have been the main reason for the premature attrition of the clinical drug development of promising new anti-malarial drug candidates, such as arterolane or artefenomel, or drug combinations including artefenomel-piperaquine and artefenomel-ferroquine.

Thus, anti-malarial drug innovation might be slowed down or even hindered by the motivation to achieve a single-dose cure. Professor White therefore advocates for maintaining three-day treatments as principal goal for new anti-malarial drug regimens similar to the current first-line ACT [12, 13].

### Is there a need for other treatment paradigms than either three-day ACT or SERCAP?

Although it is plausible that a single-dose radical cure anti-malarial drug regimen with potential to clear hypnozoites, gametocytes and have post-treatment prophylaxis would be ideal, the high attrition rate that this goal may cause for new anti-malarial combinations is worrying. Attrition has recently been observed for artefenomel combinations with either piperaquine or ferroquine, leading to a halt in the further development of this novel anti-malarial [14, 15]. As convincingly pointed out “the perfect [may thus become] the enemy of the good” [12].

One of the main concerns with the paradigm of single-dose therapy is that a single intake of a drug or drug combination needs to reliably achieve curative drug concentrations for at least six to eight days in > 95% of patients [16]. Anti-malarial drugs show a wide variability of pharmacokinetic characteristics and overall bioavailability is one of the main determinants for drug exposure. Thus, single-dose therapy necessitates an exceptionally wide therapeutic window of new anti-malarial drug candidates allowing for a single loading dose without causing significant safety or tolerability concerns [15]. In case of lower-than-average bioavailability due to an empty stomach, changes in gastric emptying or other features of acute illness, single-dose treatment may easily become ineffective. A single drug intake, therefore, possibly constitutes an uncertain and high bet for the treatment of a potentially lethal disease. Arguably, three-day ACT has been so successful as the variability of bioavailability and drug exposure have been evened out by multiple intakes of the drug leading to rather reliable overall drug exposure. A certain degree of redundancy in the proposed drug regimens may, therefore, be prudent and necessary to ensure high effectiveness in real-world settings.

### Alternatives to SERCAP as the ultimate goal of anti-malarial drug development

One reasonable approach to cost-effective anti-malarial drug development may be to systematically examine the required treatment duration for an optimal therapeutic effect in clinical phase II trials. The tested treatment duration should start with a three-day regimen as the longest acceptable standard for the treatment of uncomplicated malaria. Then, treatment duration should be incrementally decreased to a one-day regimen using pre-specified futility criteria. Importantly, such a step-down procedure of treatment duration needs to be conducted in the actual target population, which constitutes children < 5 years of age in high-transmission regions, as well as non-immune patients of all age groups in low-transmission regions. Interestingly, this development approach has recently been employed by Novartis for the development of ganaplacide and cipargamin.

Even if a new drug combination proves highly efficacious as single day, single-dose therapy in clinical trials, multiple doses of the drug during less than three days (ideally on a single day) should constitute the ultimate clinical development goal. The underlying reasoning mostly relies on considerations of the pharmacokinetic properties of single dose versus multiple dose therapies and their associated safety, tolerability, and efficacy in anti-malarial therapy.

To illustrate this, pharmacokinetic simulations were performed (R software, version 4.0.2; package ‘lnpk’) using typical population values of the pharmacokinetic parameters of the paradigmatic anti-malarial combination regimen artefenomel and piperaquine as reported by Macintyre et al. [15].

Based on this simulation, it becomes visible that total drug exposure over time is comparable when the drug is administered either as a single-dose or in two or in three split-doses with differences of < 0.3%. However, peak plasma concentrations are significantly lower in the two- and three-dose regimen compared to the single-dose intake. Peak plasma concentrations are known to be associated with early treatment related adverse drug reactions including nausea, vomiting, other gastrointestinal side effects as well as cardiac toxicity (e.g., presenting as QTc prolongation). A regimen leading to similar overall drug exposure while reducing peak concentrations is likely to be better tolerated and being safer than a single dose regimen.

**Fig. 1:**
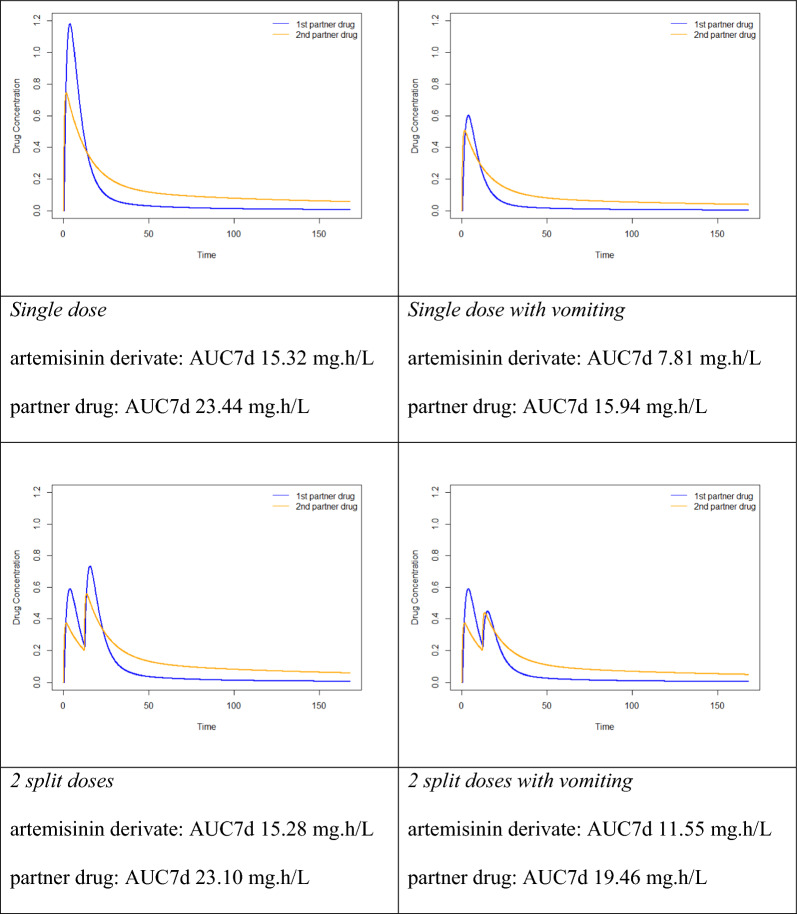

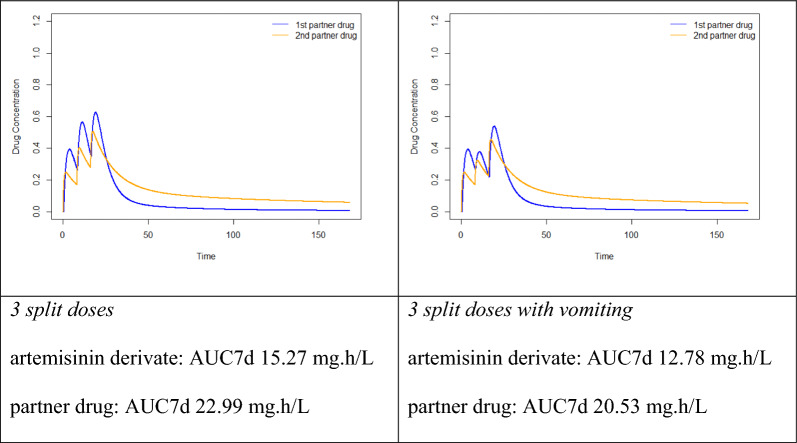
Drug concentration-time curves of single and split-dose administration of anti-malarial combination therapy with and without vomiting. AUC7d = area under the concentration-time curve day 0 to day 7

Importantly, variability of bioavailability is a major feature and problem in anti-malarial chemotherapy. Early vomiting after drug intake is a particularly frequent problem in the treatment of paediatric malaria and is associated with lower drug exposure. In the above-mentioned example, vomiting was associated with a reduction of bioavailability of 49% for artefenomel and 32% for piperaquine [15] when evaluating single dose therapy on day 7 AUC. Such a dramatically reduced total drug exposure is associated with an increased risk for treatment failure and highlights the disadvantages of a one-time opportunity to achieve sufficient drug levels. The effects of splitting the total dose of an anti-malarial into two or three doses on overall drug exposure in case of early vomiting are schematically shown (Fig. 1). AUC is a measure of overall drug exposure, which is arguably the most important marker for overall anti-malarial drug efficacy. It should reasonably well correlate with the time above minimal inhibitory concentration.

Splitting of the total dose leads to an increase in area under the curve (AUC) of the artemisinin-class derivative of 48–64% compared to the single dose regimen. When vomiting occurs after the first of two split-doses, AUC, therefore, still reaches about 75% of its average level. Importantly, considering a three-dose single day regimen of identical total dose, vomiting of the first of three split-doses leads to an AUC that is even 83% of the average AUC in patients not vomiting. This simulation clearly shows the higher resilience of multiple-dose regimens on varying bioavailability due to episodes of vomiting or other reasons for reduced drug uptake. While this simulation relies on published data of artefenomel-piperaquine combination therapy, it is of course understood that this in silico modelling provides only a theoretical framework. There is thus a need for clinical trials and field input on the suitability of a dose-splitting approach.

In summary, peak plasma concentrations are associated with early tolerability concerns and our pharmacokinetic understanding indicates that multi-dose regimens show significantly more resilience against early vomiting. Thus, multi-dose regimens potentially safeguard high efficacy and may lead to better tolerability while maintaining overall drug exposure. Similarly, multiple doses administered on one day would allow for an evening out of some variability in bioavailability caused by unobserved vomiting or feeding conditions. Even though single-day, multiple-administration treatments are not as convenient as single-dose therapies, they are still a major logistical improvement as compared to 3-day treatments and may still be associated with improved treatment adherence as most malaria patients are still suffering from symptoms during the first 24 h—thus being reminded to take the anti-malarial drug.

## Conclusions

Single-dose anti-malarial therapies seem to us as an over-ambitious goal that may more hinder than help the urgently needed clinical development of new anti-malarial drug regimens. For now, three-day artemisinin-based combination therapy remains a reliable concept in most highly malaria-endemic regions of the world. However, a shortened duration of new treatment regimens will help improving treatment adherence and, therefore, effectiveness. While the systematic assessment of three-day, two-day, and one-day regimens is welcome, SERCAP should not constitute the ultimate goal of anti-malarial drug development. Instead, funders should also encourage development of multiple-dose regimens that are ideally administered within a single day. Developing drug candidates as a multiple-dose single-day anti-malarial combination therapy will reduce attrition of new drugs while maximizing the potential benefits in effectiveness of new drug regimens.

## Data Availability

The data and graphic simulation used are available from the corresponding author on reasonable request.

## Abbreviations

ACT: Artemisinin-based combination therapy
AUC: Area under the curve
G6PD: Glucose-6-phosphate-dehydrogenase
MMV: Medicines for malaria venture
SERCAP: Single-exposure radical cure and prophylaxis
TPP: Target product profile
